# Fracture unicondylienne latérale sagittale du fémur associée à une fracture homolatérale verticale de la patella

**DOI:** 10.11604/pamj.2015.20.218.6346

**Published:** 2015-03-10

**Authors:** Youness Sasbou, Mohammed Boussaidane, Loubet Unyendje Lukulunga, Mohammed Azzouz, Youness Mhammdi, Driss Benchebba, Belkacem Chagar

**Affiliations:** 1 Université Mohammed V, Faculté de Médecine et de Pharmacie, Service de Chirurgie Orthopédique, Hôpital Militaire d'Instruction Mohammed V, 10000 Rabat, Maroc

**Keywords:** Condyle fémoral, patella, ostéosynthèse, femoral condyle, patella, osteosynthesis

## Abstract

Un patient de 24 ans a subit un traumatisme du genou droit suite à un accident de la voie publique occasionnant une fracture simultanée uni condylienne latérale et patellaire verticale. Cette association est exceptionnelle et aucun cas n'as été retrouvé dans la littérature. Un diagnostic précis clinique et radiologique suivi d'une prise en charge précoce et adaptée par une ostéosynthèse interne et une rééducation fonctionnelle ont permis d'obtenir de bons résultats à long terme.

## Introduction

Les fractures uni-condyliennes du fémur sont des lésions rares, fréquemment associées à d'autres lésions traumatiques, elles peuvent passer initialement inaperçues. Elles surviennent après un choc direct sur le genou fléchi, une réduction anatomique suivie d′une ostéosynthèse interne solide sont essentielles pour l′obtention de bons résultats à long terme. Nous rapportons un cas exceptionnel de fracture simultanée du condyle fémoral externe et de la patella

## Patient et observation

**Figure 1:**
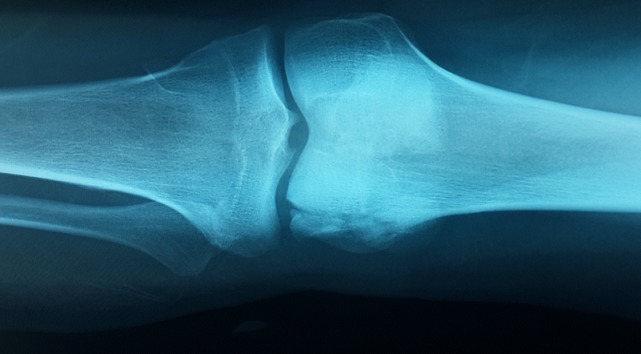
Radiographie de face du genou objectivant une fracture sagittale du condyle fémoral externe peu déplacée, associée à une fracture verticale déplacée de la patella

**Figure 2:**
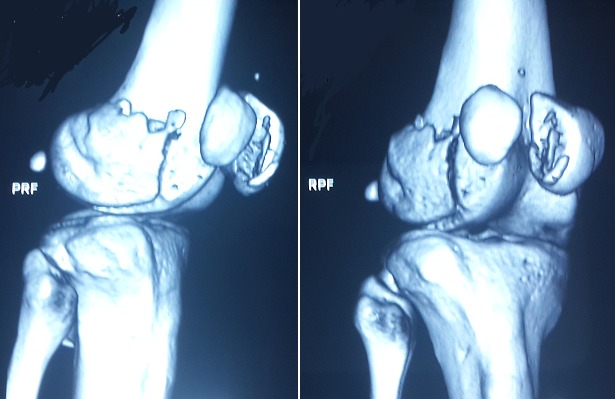
Intérêt du TDM du genou confirmant la fracture de la Rotule et du condyle fémoral

**Figure 3:**
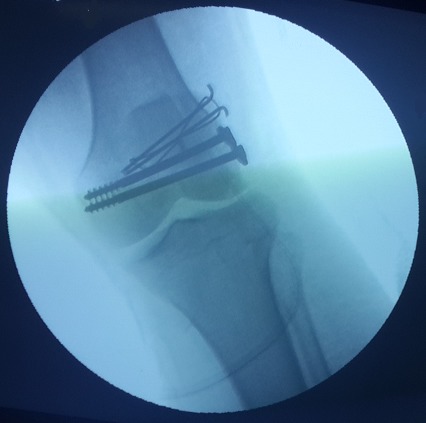
Ostéosynthèse par vissage du condyle fémoral et un embrochage haubanage de la Rotule

Il s'agit d′un patient âgé de 24 ans, sans antécédents pathologiques particuliers, qui a été admis aux urgences suite à un accident de la voie publique occasionnant un traumatisme par choc direct frontal sur le genou droit. Le patient avait présenté une douleur associée à un gonflement et une impotence fonctionnelle totale du membre inferieur droit. L′examen clinique a mis en évidence une douleur et un gonflement du genou droit à la palpation sans ouverture cutanée ni ecchymose associées, l′examen vasculo-nerveux du membre inferieur droit était normal et le reste de l′examen physique du patient était sans particularité. Des radiographies du genou droit de face et de profil ont été réalisées en urgence et ont mis en évidence une fracture sagittale du condyle fémoral externe peu déplacée, associée à une fracture verticale déplacée de la patella (Figure 1). Afin de mieux analyser ces deux fractures et de planifier le geste chirurgical, une tomodensitométrie du genou droit a été réalisée et a confirmé le diagnostic (Figure 2). Le patient a bénéficié d′un acte chirurgical avec un abord médian et une réduction de la fracture uni condylienne, suivie d′une ostéosynthèse par vissage, puis une réduction de la fracture de la patella et une ostéosynthèse par un embrochage haubanage sous control scopique per-opératoire (Figure 3). La marche sans appui a été autorisée dès le lendemain avec l′utilisation de béquilles, et un programme de rééducation fonctionnelle a été débuté après la chirurgie pour permettre au patient de retrouver une flexion complète du genou. A la 8ème semaine en post-opératoire, les deux fractures avaient consolidé sur le plan radiologique, et une marche avec appui total a été autorisée progressivement. Lors de la dernière visite du suivi, à 12 mois après l′intervention chirurgicale, le patient n′avait pas de douleur et était capable d′atteindre une mobilité normale du genou droit avec 150° de flexion et une extension complète. Les radiographies ont objectivé la consolidation des fractures.

## Discussion

Les fractures uni-condyliennes du fémur représentent seulement 0,65% de toutes les fractures du fémur [1], elles n′ont jamais été traitées en détail dans la littérature orthopédique, notamment en tant que classe de fractures [2]. Elles présentent un problème diagnostique, car elles sont souvent négligées en raison de leur association fréquente à d′autres fractures de la même région ou d′autres régions. En outre, elles sont caractérisées par une grande variabilité anatomique, une difficulté d′évaluation radiologique et une approche thérapeutique controversée [3, 4]. Elles surviennent après un impact direct, une avulsion ou une force de cisaillement sur le genou, généralement secondaires à des accidents sportifs ou de la voie publique, notamment un traumatisme de tableau de bord [5, 6]. Ces fractures intéressent le condyle fémoral latéral trois fois plus souvent que le condyle médial. Le valgus physiologique entraine une composante d′abduction, ce qui explique la fréquence accrue de fractures du condyle latéral. La zone latérale de la trochlée, étant plus large et orientée dans un plan oblique, augmente l′exposition du condyle latéral [7, 8]. Divers classifications ont été proposées, comprennent ceux d'Egund-Kolmert, Seinsheimer, Neer-Grantham-Shelton et l′AO-ASIF système. Ces fractures peuvent être traitées soit orthopédiquement par un plâtre cruro-pédieux genou en légère flexion, précédé ou non d'une traction ou chirurgicalement [9]. Dans notre cas, la fracture unicondylienne était de type B1 selon la classification de l′AO-ASIF et le patient a bénéficié d′un traitement chirurgical. Les fractures unicondyliennes peuvent êtres isolées ou associées à une fracture fémorale supra condylienne ou inter condylienne homolatérale, à une fracture du col ou de la diaphyse fémorale ou à une luxation patellaire [10]. Ce cas de fractures homolatérales simultanées, unicondylienne sagittale et patellaire verticale, est extrêmement rare. A notre connaissance, une telle association de fractures n′a pas encore été rapportée dans la littérature et seul un cas de fracture de Hoffa associée à une fracture patellaire a été rapporté. Dans notre cas, on pense que le mécanisme de ces fractures simultanées résulte de la combinaison entre les forces du traumatisme direct causant la fracture unicondylienne, et éventuellement la contraction musculaire brutale du quadriceps provoquant la fracture verticale de la patella. L'examen physique révèle généralement un œdème, un épanchement, ou des lésions cutanées dans la région du genou, l′examen neurovasculaire du membre doit être soigneusement effectué. Le diagnostic radiographique sur des radiographies standards peut être difficile, et des incidences radiographiques antéro-postérieures, latérales et patellaires tangentielles, sont nécessaires pour confirmer le diagnostic et préciser les caractéristiques de ces fractures [11]. Une tomodensitométrie est nécessaire pour décrire de façon plus précise la fracture condylienne ainsi que les fractures éventuellement associées [12]. Concernant l′attitude thérapeutique, le traitement chirurgical est le plus recommandé, il consiste en une réduction à foyer ouvert suivie d′une stabilisation par différentes techniques, telles que des plaques, des vis condyliennes spongieuses, vis canulées, vis de Herbert, vis Barr, ou vis-plaque. Dans notre cas l′ostéosynthèse de la fracture unicondylienne a été réalisée par vissage (deux vis canulées 6,5), et celle de la fracture patellaire a été réalisée par un embrochage haubanage.

## Conclusion

La fracture uni-condylienne sagittale est une lésion rare, et son association avec une fracture patellaire verticale comme notre cas est exceptionnel. Le mécanisme est généralement un traumatisme à haute énergie. Ces fractures doivent être traitées chirurgicalement avec une réduction anatomique à foyer ouvert et une ostéosynthèse interne stable suivie d′une rééducation fonctionnelle précoce afin d′obtenir de bons résultats fonctionnels à long terme.
